# Porcine Adipose Tissue-Derived Mesenchymal Stem Cells Retain Their Proliferative Characteristics, Senescence, Karyotype and Plasticity after Long-Term Cryopreservation

**DOI:** 10.1371/journal.pone.0067939

**Published:** 2013-07-09

**Authors:** Rafael Dariolli, Vinicius Bassaneze, Juliana Sanajotti Nakamuta, Samantha Vieira Omae, Luciene Cristina Gastalho Campos, Jose E. Krieger

**Affiliations:** Heart Institute (InCor), University of São Paulo Medical School, São Paulo, São Paulo, Brazil; University of Sao Paulo - USP, Brazil

## Abstract

We and others have provided evidence that adipose tissue-derived mesenchymal stem cells (ASCs) can mitigate rat cardiac functional deterioration after myocardial ischemia, even though the mechanism of action or the relevance of these findings to human conditions remains elusive. In this regard, the porcine model is a key translational step, because it displays heart anatomic-physiological features that are similar to those found in the human heart. Towards this end, we wanted to establish the cultural characteristics of porcine ASCs (pASCs) with or without long-term cryostorage, considering that allogeneic transplantation may also be a future option. Compared to fresh pASCs, thawed cells displayed 90–95% viability and no changes in morphological characteristics or in the expression of surface markers (being pASCs characterized by positive markers CD29^+^; CD90^+^; CD44^+^; CD140b^+^; CD105^+^; and negative markers CD31^−^; CD34^−^; CD45^−^ and SLA-DR^−^; n = 3). Mean population doubling time was also comparable (64.26±15.11 hours to thawed cells vs. 62.74±18.07 hours to fresh cells) and cumulative population doubling increased constantly until Passage 10 (P10) in the entire cell population, with a small and gradual increase in senescence (P5, 3.25%±0.26 vs. 3.47%±0.32 and P10, 9.6%±0.29 vs. 10.67%±1.25, thawed vs. fresh; SA-β-Gal staining). Chromosomal aberrations were not observed. In addition, under both conditions pASCs responded to adipogenic and osteogenic chemical cues *in vitro*. In conclusion, we have demonstrated the growth characteristics, senescence, and the capacity of pASCs to respond to chemical cues *in vitro* and have provided evidence that these properties are not influenced by cryostorage in 10% DMSO solution.

## Introduction

Adult mesenchymal stem cells have been investigated in pre-clinical approaches for tissue regeneration [1]–[6]. The adipose tissue is an important source due to large and easily accessible body reservoirs. Adipose tissue-derived mesenchymal stem cells (ASCs) were first isolated in humans [7] and later from various species, including rodents [8], [9] and swine [10]. These cells show low levels of immunogenicity and have immunomodulatory properties [1], [6], [11], [12] and may be useful for allogeneic transplantation.

We and others [2], [4], [13] have shown that ASC can mitigate cardiac functional deterioration after myocardial ischemia in rodents, which appears to be associated with paracrine effects related to these cells in an ischemic environment rather than the earlier considered transdifferentiation events [2], [4], [14], [15].

Rodent models are frequently and efficiently used to determine the response and potential mechanism of action underlying novel cardiovascular therapeutic approaches. However, extrapolation of these types of data directly to humans is limited because of the specific structural and pathophysiological response associated with the model [16]. In this regard, the porcine model displays heart anatomic-physiological features that are similar to those found in the human heart, but the specific characteristics of porcine ASCs (pASCs) culture and the influence of long-term cryostorage on these cells is poorly understood.

The precise cell number required for therapeutic approaches has not been established, but one may anticipate that for autologous transplantation some level of expansion will be necessary. This issue may also be confounded if one contemplates allogenic transplantation with cells cultured for several passages and available off the shelf after long-term cryostorage, which may be associated with nuclear aberrations [17], [18].

Thus, we wanted to determine the *in vitro* cell culture characteristics of pASCs before and after cryopreservation. Our findings provide evidence that cell culturing under the experimental conditions described and long-term cryopreservation of pASCs are not associated with increased senescence or morphological characteristics, which is consistent with the idea that off-the-shelf approaches are feasible.

## Materials and Methods

### Isolation, Expansion, and Freezing of pASCs *ex vivo*

Subcutaneous adipose tissue was obtained by surgical procedures from adult male pigs (MS60 EMBRAPA lineage), after anesthesia and asepsis procedures. pASCs were isolated from 300 g of adipose tissue as previously described [2], [19], cultivated in DMEM-Low supplemented with 10% FBS. On average, 10 grams of adipose tissue yielded 7 million cells at passage 0, promoting a quick and easy generation of a porcine stem cell bank. Cells were expanded in culture until P4 and frozen in a solution with 10% dimethyl sulfoxide (DMSO≥99.5% pure; Sigma) in FBS, for long-term storage (3.3×10^6^ cells/vial in a final volume of 1 mL, temp −196°C). All the characterization assays were performed on pASC cryopreserved for at least 3 and even 12 months. This protocol was approved by the institutional CAPPesq Ethics Committee (Protocol #022/09).

### Flow cytometry analysis – Viability (Annexin/PI assay)

The cell viability was assessed by flow cytometry Annexin/PI assay in pASC of three different animals. The stain of the pASCs was performed according to the manufacturer’s instructions of the kit Annexin V: FITC Apoptosis Detection Kit II (Becton Dickinson; BD Pharmingen™) and then the samples were subjected to flow cytometer FACSCalibur (Becton Dickinson, San Jose, CA, USA). A total of 10,000 events were acquired on a flow cytometer, and Cell Quest software (BD Biosciences) was used for further analysis.

### Flow cytometry analysis – Phenotypic Markers

The pASCs immunophenotype was analyzed by flow cytometry using the flow cytometer FACSCalibur (Becton Dickinson, San Jose, CA, USA). Cells were harvested after being washed twice with PBS. In brief, 0.5×10^6^ cells, at passages 4 and 5, were incubated for 30 minutes at 4°C with purified antibodies CD29 (1∶50 dilution; 552369), CD90 (1∶20; 555593), CD44 (1∶50; 559250), and CD31 (1∶50 555025) (BD Biosciences, San Jose, CA), and further incubated for 30 minutes at 4°C with secondary antibody (Alexa-488, Invitrogen). A total of 10,000 events were acquired on a flow cytometer, and Cell Quest software (BD Biosciences) was used for further analysis.

### RT-PCR

Total RNA was extracted by the single-step method using Trizol reagent (Invitrogen, Carlsbad, CA) according to the manufacturer’s instructions. cDNA synthesis from total RNA (1 µg) was produced by reverse transcription (RT) using the superscript II kit according to the manufacturer’s protocol (Invitrogen). Polymerase chain reaction (PCR) was performed to test the primers, using pig fibroblasts and endothelial cells of mammary artery cDNAs as template and the reactions were performed using Taq-polymerase manufacturing protocol (Promega). Primer sequences and expected product lengths are shown in Table S1.

### pASC Doubling Time (DT)

To evaluate population growth, the protocol described by Freshney was used [20]. The DT calculation was determined by DT = [Log_10_ (2x) – Log_10_ (x)]/A, where A is the linear regression coefficient expressed in hours. The experiment was performed from passages 5 to 10 (P5 to P10).

### Cumulative Population Doubling

pASCs cumulative population doubling (CPD) was calculated as previously described [20]. pASCs were maintained in DMEM supplemented with 10% FBS until 80% confluence. The cells were detached with 0.5% trypsin-EDTA and re-plated at 1×10^4^ cells/cm^2^. The CPD was calculated by [ln (N2/N1)/ln2] where *N2* are the cells (*x 10^4^*) recovered from a seeding of N1 cells (*x 10^4^*). The CPD was determined from P5 to P10.

### Senescence

SA-β-Gal staining at pH 6.0 was performed as described earlier [21]. Positive controls were prepared using pASCs at P5, treated with 200 and 400 µM H_2_O_2_ for 2 h at 37°C, and cultured for 8 days in fresh medium. Next, cells were plated onto 35-mm culture plates at 5×10^4^ cells/well and SA-β-Gal staining was performed.

### Karyotype

Cells were incubated with 10 µg/ml colcemid (Gibco) for 90 minutes in humidified incubator (5% CO2, 37°C) and then detached. The pelleted cells were incubated in 5 ml of hypotonic solution (0.1 M KCl) for 18 min at 37°C followed by fixation with methanol/glacial acetic acid (3∶1) solution. Fixed cells were dropped on wet slides and air-dried per 3 days to obtain standard G-banding chromosome pattern. *Giemsa* staining was carried out as indicated by manufacturers (KaryoMAX® Giemsa, Gibco). Metaphases were fully karyotyped under a Leica HC microscope. Images were then captured with digital camera Leica DC250 and using Leica CW4000 Karyo software.

### Plasticity of pASCs

pASCs at passage 5 were analyzed for their capacity to differentiate toward the adipogenic and osteogenic lineages. To induce differentiation, cells were cultured with specific induction media as described by Zuk and colleagues [7]. The pASCs were plated at a density of 1×10^4^ cells/cm^2^ and experimental manipulations were started when the plates reached 80% confluence (2–3 days). Further, they were examined with Oil Red O (Sigma) and Alizarin Red S (Sigma) staining [22].

### Statistical Analyses

Results are expressed as mean ± standard error of the mean (SEM). One- or two-way analysis of variance (ANOVA) with Bonferroni post-hoc test or the unpaired Student *t* test was utilized to compare groups, as appropriate. All statistical analyses were performed using GraphPad Prism 5.0 (GraphPad Software Inc., CA, USA). p values <0.05 were considered significant.

Further detailed information is presented in the Materials and Methods S1.

## Results

### Isolation, Expansion, Storage and Viability of pASCs after Thawing

**Figure 1:**
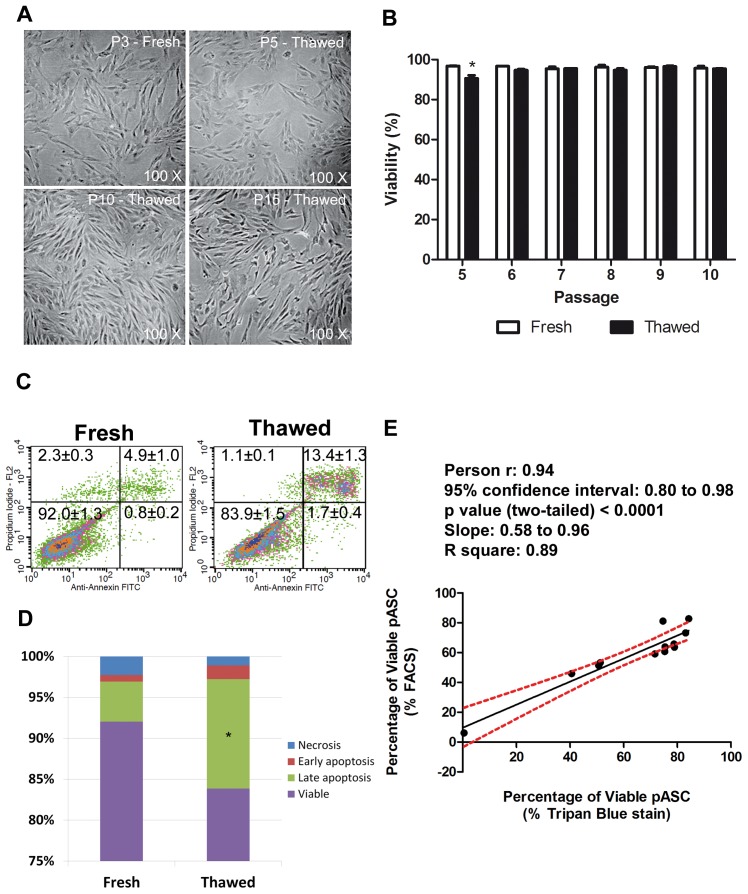
pASC morphology and viability are maintained after long-term cryostorage. Morphological profiles for pASCs **A)** after passage 3 (before freezing) and passages 5, 10, and 15 (after thawing). Note similar fibroblast–like morphology in all represented passages. **B)** Quantification of viability of pASCs by Tripan blue dye assay in Passage 5 after thawing displayed a small but significant reduction versus fresh cells (P6–P10; *p<0.001; n = 3). **C)** Representative FACS plot showing the viability of P5 pASC by Annexin/PI assay (mean%±SEM; n = 3). **D)** Quantification of cell death by type (*p<0.0001 fresh late apoptotic vs. thawed late apoptotic cells; n = 3). Correlation curve of percentage of viability between Tripan blue dye assay×Annexin/PI assay.

pASCs were extracted and their population characteristics and ability to acquire adipose and osteogenic phenotype features were explored. pASCs demonstrated a high capacity for expansion *in vitro* and exhibited fibroblast-like morphology (Figure 1A). No change in cell viability was observed in fresh versus thawed cells using Trypan Blue vital dye to assess viability. Thawed pASCs provided about 90–95% viable cells from P5–P10 (90.58%±1.52 in P5 vs. 95.40%±0.26 viable cells in P6–P10, respectively) (Figure 1B). Similarly, the cell viability remained high and stable through multiple passages for fresh cells (96.18%±0.27; Figure 1B). Porcine ASCs in passage 5 were also subjected to annexin/PI assay to measure kinds of cellular death. Essentially the data showed that the increase of cell death after thawing was linked to the cycles of storage/re-expansion (Late Apoptosis: 13.37±1.35 vs. 4.89±1.07; mean±SEM; Thawed vs. Fresh, n = 3; Figure 1C and D). Furthermore, P5 pASCs subjected to controlled induced death by different doses of ultra-violet light showed a positive correlation curve comparing Tripan blue and Annexin/PI assesments (Figure 1E).

### Cryopreservation does not Influence the Expression of Mesenchymal Surface Markers

**Figure 2:**
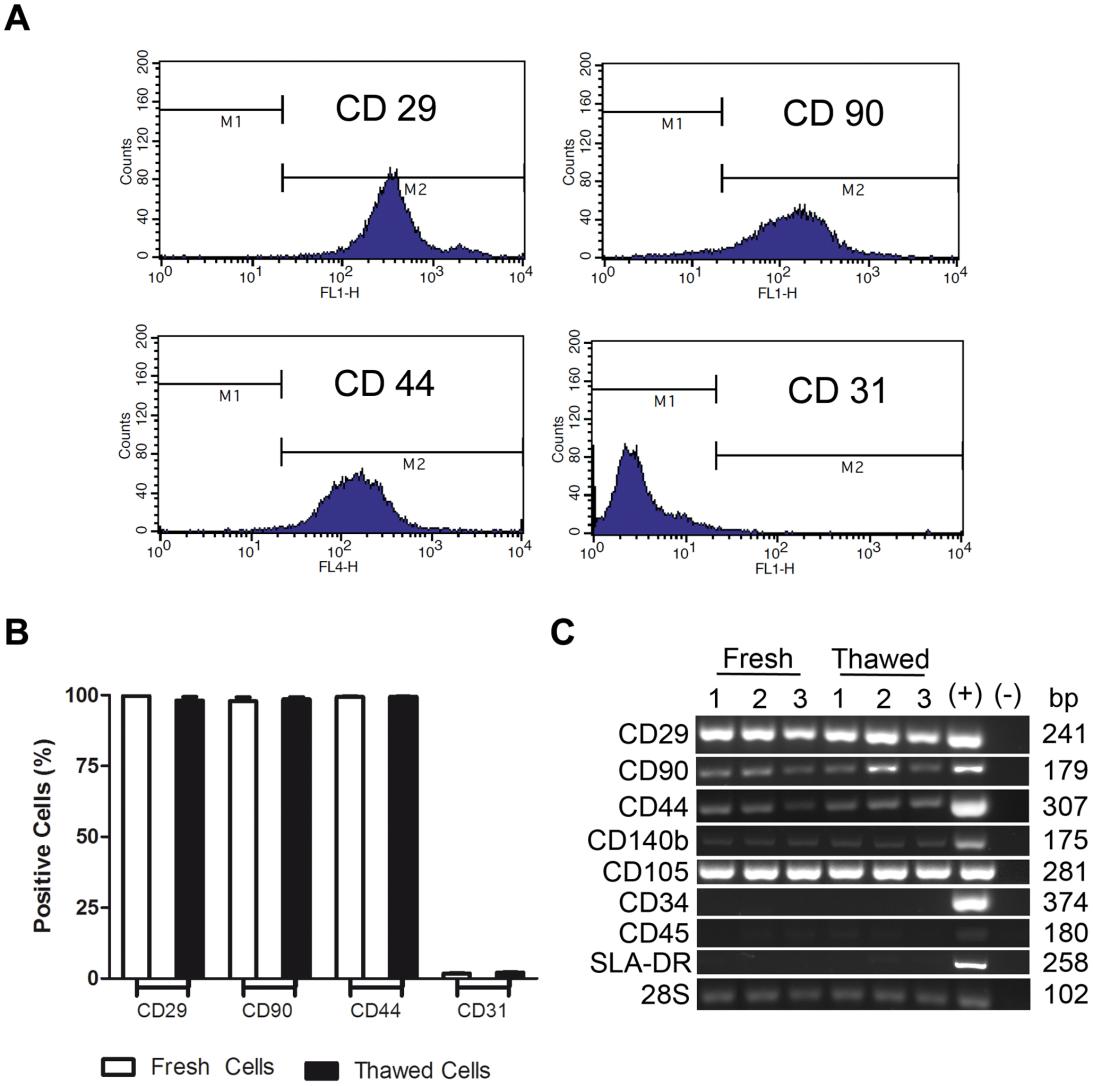
Cryostorage does not influence the expression of mesenchymal surface markers. **A)** FACS histogram representations for each analyzed surface marker in pASCs. **B)** Quantification of surface markers (means expressed as percentages; n = 3). **C)** Representative semi quantitative RT-PCR comparing fresh and thawed cells by mRNA expression for positive and negative phenotypic markers (2% agarose gel). Numbers 1, 2 and 3 are relative to different animals used to extract the pASCs.

Cultured pASCs from P4 to P5, expressed mesenchymal surface markers CD29^+^ (99.74%±0.10; n = 3), CD90^+^ (97.84%±1.32; n = 3), and CD44^+^ (99.39%±0.19; n = 3) and the endothelial cell marker CD31^−^ (1.76%±0.21; n = 3) (Figure 2A and B and Table 1). Furthermore, RT-PCR confirmed the FACS analysis and added that the pASC were positive for CD140b and CD105 markers and negative for CD34, CD45 and SLA-DR markers (Figure 2C). So, pASCs were characterized as positive for CD29, CD90, CD44, CD140b, CD105, and negative for CD31, CD34, CD45 and SLA-DR (Figure 2). Similar results were obtained in pASC samples that did not undergo cycles of storage/re-expansion (Table 1 and Figure 2B and C).

**Table 1:** pASC immunophenotypic characterization before and after freezing**.**

Pig	1		2		3		Mean		
Cell condition	F	T	F	T	F	T	F±SE	T±SE	Total±SE
**CD29 (+)**	95.8	99.8	98.9	99.5	99.8	99.9	98.1±1.2	99.7±0.1	98.9±0.7
**CD90 (+)**	97.0	95.2	99.5	99.1	99.1	99.2	98.5±0.8	97.8±1.3	98.2±0.6
**CD44** **(+)**	99.5	99.0	99.6	99.7	99.4	99.4	99.5±0.1	99.4±0.2	99.4±0.1
**CD31 (-)**	1.8	1.3	2.3	2.0	2.3	1.9	2.1±0.2	1.8±0.2	1.9±0.2

**Abbreviations:** F = fresh cells; D = thawed cells; 1.2 and 3 =  number of pigs evaluated; SE = standard error.

### pASC Growth Kinetics after Cryopreservation

#### Thawed pASCs had a similar population doubling time versus fresh cells

**Figure 3:**
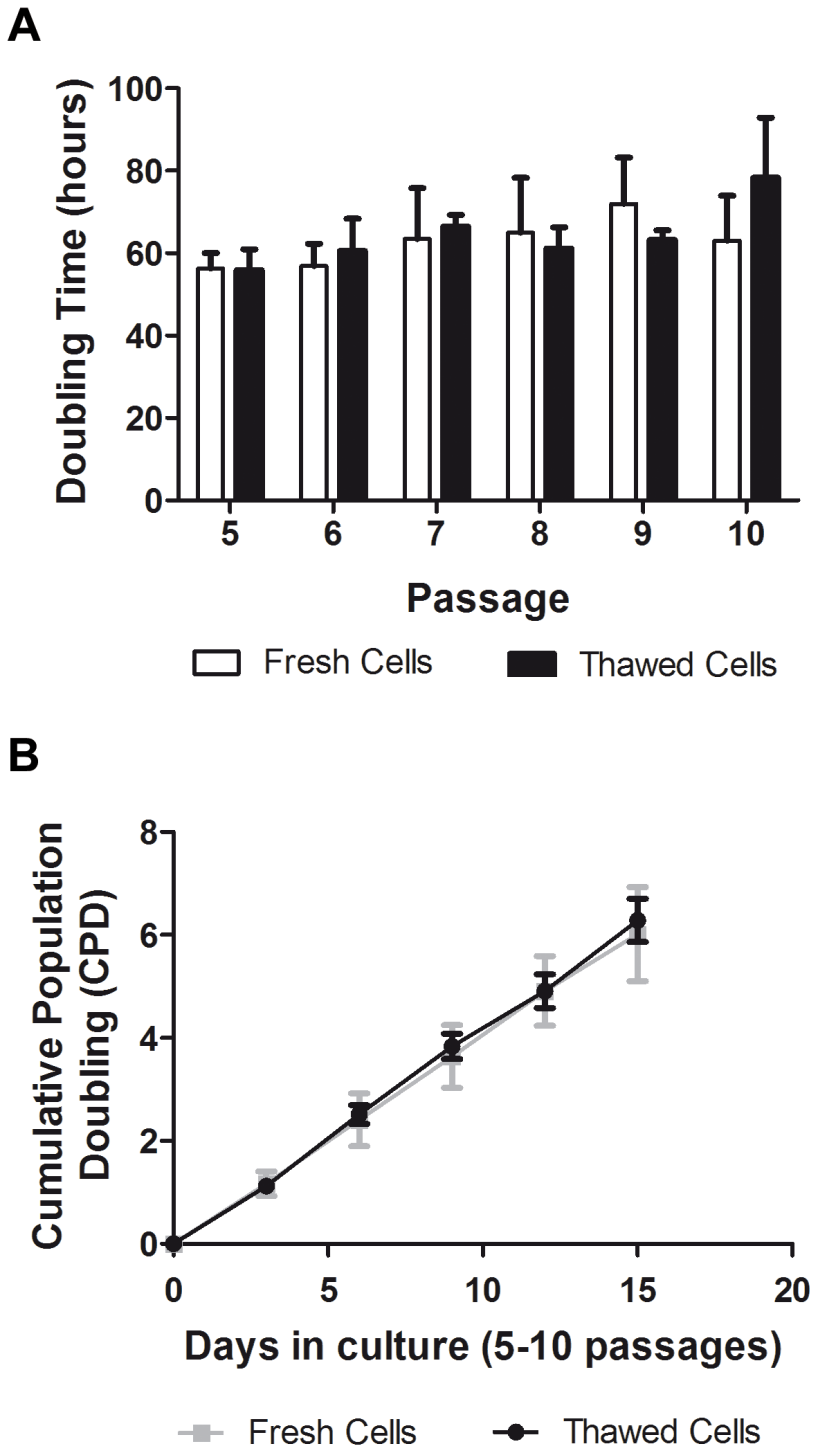
Growing kinetics are similar for thawed and fresh pASC. **A)** There were no significant differences in doubling times between passages in different conditions (64.26±15.11 hours to thawed cells vs. 62.74±18.07 hours to fresh cells; p>0.05; n = 4). **B)** pASCs cumulative population doubling (CPD) indicates a relatively constant population doubling rate between thawed and fresh cells from Passage 5 to 10. (thawed cells CPD mean = 1.27 vs. fresh cells CPD mean = 1.20; p>0.05. n = 4).

pASC doubling time in thawed cells during the proliferative phase - P5 to P10 - was 64.26±15.11 hours, and this remained unchanged throughout passage (Figure 3A). In addition, these data were similar for the fresh pASC populations (62.74±18.07 hours; Figure 3A).

#### Cumulative population doubling is constant and similar between thawed and fresh pASCs

The average cumulative population doubling (CPD) assessed from P5 to P10 was 1.27 in thawed cells. A linear relationship between CPD and passage number was observed, indicating a relatively constant population doubling rate over the range studied (y = 1.240 x +5.023, where y = cumulative doubling time and x passage, R^2^ = 0.9996, Pearson’s r = 0.9998; Figure 3B). Furthermore, no significant reduction in CPD was observed, suggesting that frozen pASCs maintained their proliferative potential during the study period. These assessments were performed in cells not exposed to storage, but similar results were observed for cells undergoing storage/re-expansion cycles (mean CPD = 1.20; Figure 3B).

#### Cryostorage does not accelerate senescence or generate chromosomal aberrations

**Figure 4:**
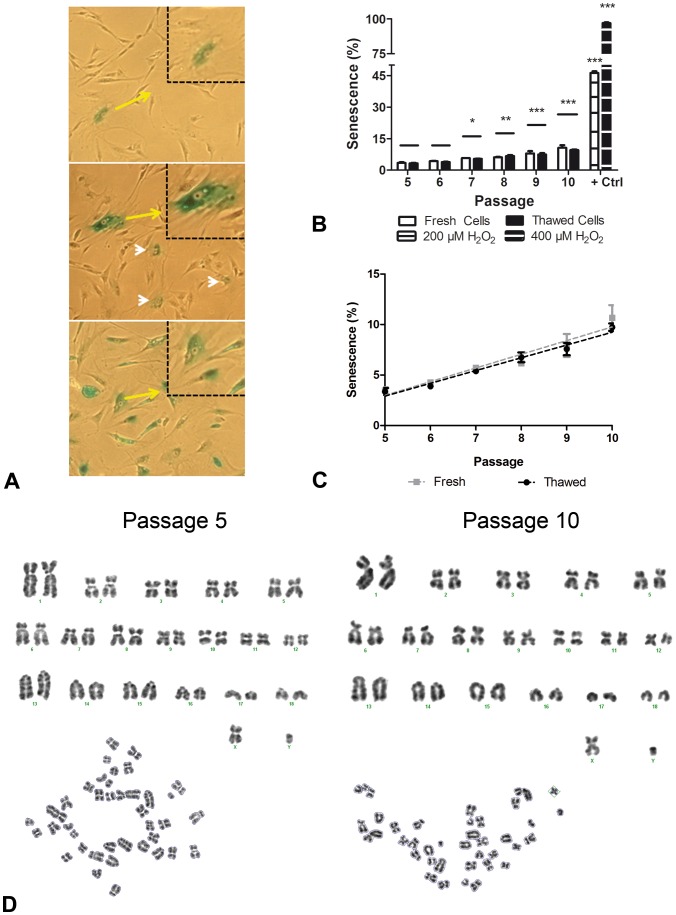
Senescence rate in pASC is not influenced by cryostorage. **A)** pASCs senescence assay results for passages 5, 10 and pASCs passage 5 positive control (400 µM H_2_O_2_) respectively. Note the elevation in green-labeled cells in passage 10 vs. 5 (white head arrows); yellow arrows indicate senescent cells. **B)** Senescence quantification for passages 5 vs. P7 * = p<0.05; P5 vs. P8 ** = p<0.01; P5 vs. P9. 10 and positive controls *** = p<0.001. P5 vs. P6 p>0.05. No significant differences were observed between fresh and thawed cells in each analyzed passage (p>0.05). **C)** Linear regression showing that the mean number between thawed and fresh senescent cells increased gradually during Passages 5–10 (Pearson’s r: fresh = 0.9694. thawed = 0.9895; R squared: fresh = 0.9397. thawed = 0.9792; p<0.001; linear regression - p slope>0.5). **D)** Representative P5 and P10 karyotypes and metaphases. No chromosomal aberrations were observed in pASC after thawing (P5) or after long-term cultivation (P10).

We also examined cell senescence from P5 to P10 by SA-β-Gal staining (Figure 4). Only 3.25%±0.26 of thawed pASCs were senescent at P5. A gradual and significant increase to positive senescence stain was observed from P5 to P10 (Pearson r = 0.9895; R squared = 0.9792; p<0.001; linear regression - p slope >0.05; Figure 4C), but senescence did not exceed 10% at P10 (Figure 1B). All this methodology was performed in fresh cells and similar results were observed (Figure 4). Furthermore, we evaluated a chromosomal profile of pASC after thawing in passage 5 and after long-term cultivation (P10). No chromosomal translocation, deletion or extra-chromosomes number were observed (Figure 4D).

### Plasticity of pASCs

To assess whether the pASCs in culture retained some degree of plasticity and cell multilineage capacity, thawed cells were treated with defined culture medium supplementation to induce differentiation toward adipogenic and osteogenic lineages [7]. Differentiation was evaluated using staining-specific protocols [22] and compared to pASCs maintained in standard medium (negative control). It is important to emphasize, however, that tests performed in different passages (5–8), with cells extracted from the same animal and using standard experimental protocol [7] still resulted in heterogeneous differentiation responses.

**Figure 5:**
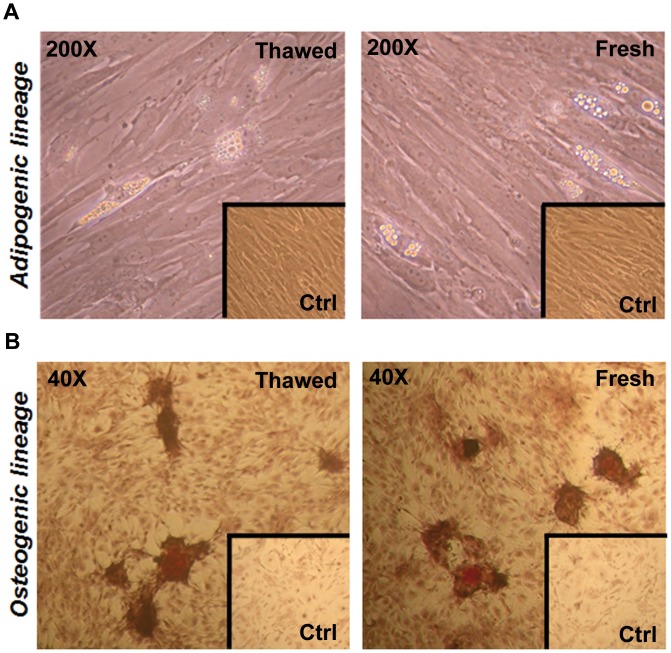
Cryostorage does not influence the plasticity of pASCs. **A)** pASCs adipogenesis induced by supplemented medium during 3 weeks in culture. **B)** pASCs osteogenesis induced by supplemented medium after 3 weeks in culture after Alizarin Red S staining.

Culturing of pASCs in adipogenic inductive medium for 3 weeks resulted in the expected increase in intracellular lipid droplets, identified under phase-light microscope (Figure 5A), but only in specific cell niches in the culture plates. No droplets were observed in positive control cells (Figure 5A, lower right inset in both fresh and thawed cells). The observed morphology of differentiated pASCs was similar to that observed for hASCs [7], [19] and others [10].

pASCs cultured in osteogenic inductive medium for 3 weeks and stained with Alizarin Red S showed cells covered with calcium deposits, consistent with the idea that the cells retained the potential to undergo osteogenesis (Figure 5B), while pASCs cultured in the control medium remained unchanged (Figure 5B, lower right inset in both fresh and thawed cells). pASCs that did not undergo storage/re-expansion cycles demonstrated similar results (Figure 5A and B).

## Discussion

The results described demonstrate that growth and senescence characteristics in pig ASC are not influenced by large-scale extraction and cryostorage. In two weeks, at P4, 3 billion of CD29^+^, CD90^+^, CD44^+^, CD140b^+^, CD105^+^ and CD31^−^, CD34^−^, CD45^−^ and SLA-DR^−^ cells can be obtained from 300 g of adipose tissue. In the first passages (P0–P3) the pASC cultures demonstrated qualitative heterogeneous morphology including endothelial-like cells and erythrocytes. On P4, the cultured cells are more homogeneous, displaying a fibroblast-like morphology consistent with the appearance of mesenchymal stem cells derived from adipose tissue of rodents [2] and humans [19].

The cryostorage procedure in the presence of DMSO, which can affect Oct-4 expression and the pluripotent potential of embryonic stem cells [23], intracellular release of Ca2+ [24] and others [25] does not appear to negatively influence pASCs undergoing the thawing process associated with long-term cryostorage. After thawing, pASC viability was higher than 90% for all tested vials (Figure 1B), similar to data obtained for cells from other species [26]–[28] and the cell death was mainly associated to late apoptosis i.e., pASCs are healthy even after a cycle of storage and re-expansion (Figure 1C and D). In addition, morphological characteristics (Figure 1A) or surface marker expression (Figure 2 and Table 1) of pASCs were not altered.

pASC growth kinetics from P5 to P10 were homogeneous and constant. The doubling time was calculated based in pASC growth curves [20] generated from controlled experiments. These cells showed a linear relationship between cumulative population doubling and passage number. These data indicate a relatively constant population doubling rate over P5–P10, and less than 3.5% of pASCs displayed senescent phenotype at P5 similar to fresh pASCs as demonstrated earlier for fresh cultured cells [18].

pASC adipogenic induction was associated with lipid droplet accumulation, although smaller and more scant than the pattern observed in hASC (data not shown), also described earlier in the literature [7], [19]. The osteocyte induction treatment resulted in pASCs with mineralized nodules stained by Alizarin Red S as previously observed in the swine model [10], [29] and hASCs [7], [19]. It is important to emphasize that both differentiation events were not uniform and were observed only in specific areas of the plate (Figure 5). Furthermore, only part of the induction attempts resulted in successful differentiation (20 and 40% for the osteogenic and adipogenic stimuli, respectively). This phenomenon may be explained, at least in part, by a variable ability of the cells to differentiate within the same cell population [30].

Our data provided evidence that pASC cell viability, morphology; growth characteristics, senescense, karyotype and capacity to respond to chemical cues are not influenced by long-term cryostorage processes using 10% DMSO. These findings are consistent with the data reported by Oak and colleagues on porcine bone marrow tissue-derived mesenchymal stem cells (pMSCs) [31].

The relevance of these findings relies on the significant observation from our laboratory and that of others using the rodent model in which ASC transplantation post-myocardial infarction was associated with amelioration in cardiac structure and function and the importance of the swine model to assess the potential of this intervention in humans, especially considering the possibility of stem cell banking readiness and off-the-shelf use of ASC for therapeutic applications.

## Supporting Information

Table S1 **Primer sequences, primer melting temperature and expected product lengths.**
(DOCX)

Materials and Methods S1 (DOCX)
