# Supplementary material for: Porcine Adipose Tissue-Derived Mesenchymal Stem Cells Retain Their Proliferative Characteristics, Senescence, Karyotype and Plasticity after Long-Term Cryopreservation
Source: PLoS One. 2013 Jul 9;8(7):e67939. doi: 10.1371/journal.pone.0067939 (PMC3706624; doi:10.1371/journal.pone.0067939)
Supplement: Table S1 — Primer sequences, primer melting temperature and expected product lengths. (DOCX) [file pone.0067939.s001.docx]

***Supporting Information***

***Table S1:*** Primer sequences, primer melting temperature and expected product lengths.

| **Gene** | **Forward 5' - 3'** | **bp** | **Reverse 5' - 3'** | **bp** | **Tm °C** | **Amplicon (pb)** | |
| --- | --- | --- | --- | --- | --- | --- | --- |
| **CD29** | GGACGCTTACTGCAGGAAAGAG | 22 | ACAGTCACAKGCRCTGCCAGTG | 22 | 56 | | 241 |
| **CD90** | CCGCTCTCGAACCAACTTCT | 20 | TTATGCCCCCACACTTGACC | 20 | 60 | | 179 |
| **CD44** | CATCTTCCACACCCACCTTC | 20 | ACCTCCTCCGACTGTTGAC | 19 | 55 | | 307 |
| **CD140b** | TACGTGCCCATGYTGGACATG | 21 | TGGTAGCTGAAGCCCACGAG | 20 | 54 | | 175 |
| **CD105** | CGCTTCAGCTTCCTCCTCCG | 20 | CACCACGGGCTCCCGCTTG | 19 | 56 | | 281 |
| **CD34** | ACCACCGTAGCCATCTCAG | 19 | CCATTGTCCTTCTTAAACTTCTCG | 24 | 55 | | 374 |
| **CD45** | GCATCCATCCTCGTCCACTGC | 21 | GATAGATGCTGGCGATGATGTC | 22 | 54 | | 180 |
| **SLA-DR** | GTGTGCGACGGAATCTATAAC | 21 | GAGCATGAGCCCTAAGAGAC | 20 | 60 | | 258 |
| **28S** | TCATCAGACCCCAGAAAAGG | 20 | GATTCGGCAGGTGAGTTGTT | 20 | 60 | | 102 |

***Abbreviations:*** *bp = base pars; Tm = Melting Temperature.*
