# Supplementary material for: Porcine Adipose Tissue-Derived Mesenchymal Stem Cells Retain Their Proliferative Characteristics, Senescence, Karyotype and Plasticity after Long-Term Cryopreservation
Source: PLoS One. 2013 Jul 9;8(7):e67939. doi: 10.1371/journal.pone.0067939 (PMC3706624; doi:10.1371/journal.pone.0067939)
Supplement: Materials and Methods S1 — (DOCX) [file pone.0067939.s002.docx]

***Supporting Information***

***Materials and Methods***

1. ***Isolation, expansion and cryostorage pASC ex vivo***

Subcutaneous adipose tissue was obtained by surgical procedures from four (1 pig - 300 g and another 3 pigs 30-50 g) young adult male pigs (MS60 EMBRAPA lineage), after anesthesia and asepsis procedures.

In laminar flow cabinet, the adipose tissue was mechanically digested and then the adipose tissue was dissociated by enzymatic digestion (collagenase type 1A 0.075% in Free DMEM - C-2674, Sigma ®, St. Louis, MO) and centrifuged. The pelleted cells were then recovered and plated onto 10-cm culture plate (NUNC, Rochester, NY). Plating and expansion medium consisted of Dulbeccos’ modified Eagle medium (DMEM) low glucose with 10% fetal bovine serum (FBS) and penicillin/streptomycin antibiotics (Invitrogen Corporation, Carlsbad, CA). After 24-72 hours, cultures were washed in PBS to remove remaining erythrocytes and other unattached cells. Cells were maintained at 37°C with 5% CO_2_ in tissue culture dishes or flasks (Becton Dickinson, Franklin Lakes, New Jersey) until they reached 80% of confluence (usually within 2-3 days). Once 80% confluent (P = 0), cells were detached with 0.5% trypsin-EDTA (Cultilab, São Paulo, SP, Brazil) and either re-plated at 1×10^4^cells/cm². On average, 10 grams of adipose tissue yielded 7 million cells at passage 0, promoting a quick and easy generation of a porcine stem cell bank (900 vials about 3 billion cells – with only one animal). Cells were expanded in culture until P4 and frozen in a solution with 10% dimethyl sulfoxide (DMSO ≥99.5% pure; Sigma) in FBS (900 µl of FBS to 100 µl of DMSO). The cells (900 µl of FBS) were inserted in vials and then 10% of DMSO was added. Vials were subjected to careful homogenization and placed on ice not exceed 10 minutes then the vials were placed in dry ice at least 10-20 minutes. After this, the vials were placed into ultrafreezer -86°C (Thermo^®^) no more than 3 days and finally transferred to liquid nitrogen (-196°C).

To perform the thawing of cells the vials were placed on ice and at least 10 mL of fresh and cold FBS was rinsed until the cell plus DMSO solution was completely thawed. The solution was centrifuged per 5 minutes, 4°C and 1500RPM and then the pellet of cells was homogenized in 5 mL of 10% FBS - DMEM-Low. Cells were counted in Neubauer chamber and plated at 1×10^4^cells/cm² in culture dishs needed for each experiment.

Three other animals were used for comparative characterization of pASCs stem cell bank.

1. ***Flow cytometry analysis – Viability (Annexin/PI assay)***

To perform the staining with Annexin V and propidium iodide (PI) the manufacturer’s instructions of the kit Annexin V: FITC Apoptosis Detection Kit II (Becton Dickinson; BD Pharmingen™). In brief, each 1 million pASC (fresh detached of the culture dish or thawed of the vial) were washed twice with cold PBS 1X. The cells were pelleted in 50 mL tubes (centrifuged 1500 RPM, 5 min, 4°C) and then re-suspended in 1X Binding Buffer at a concentration of 1 x 10^6^ cells/ml. Aliquots with 100 µl of the solution (1 x 10^5^ cells) were transferred to 5 ml cytometry tubes and then added 5 µl of FITC Annexin V and 5 µl PI. The tubes were gently vortex and incubated for 15 min at RT (25°C) in the dark. After this was added 400 µl of 1X Binding Buffer to each tube and the cells were analyze by flow cytometry.

To perform a good test, positive and negative controls were established. To obtain the standard profile of pASC was used a tube no stained. Other tube was marked only with Annexin V and another tube stained only with PI. Positive controls were established using pASCs subjected to ultra-violet light in different intensities (100, 200, 300-1000 and 2000 millijoules/cm^2^– UV Stratalinker® 1800; Stratagene). These cells were subjected to both, Tripan blue dye stain followed by viable cell count in Neubauer chamber and to Annexin/PI assay. With these two data was generated a correlation curve which can be seen in the original article (Figure 1E).

1. ***RNA Isolation and Reverse Transcriptase Polymerase Chain Reaction (RT-PCR)***

To phenotypic markers assessment pASCs were cultured in standard conditions (10% FBS DMEM-Low). When in passage 5 about 1 million of cells (1x 100 mm culture plate) were subjected to 1 mL Trizol reagent (Invitrogen, Carlsbad, CA) in fresh conditions and after cycle of storage/re-expansion. First the plates were washed twice with PBS 1X. The PBS was discarded and the plate was shaken quickly to dry and then the Trizol was added. Using a scrapper the cells were detached, collected and stored in ultrafreezer (-86°C).

Total RNA was extracted by the single-step method using Trizol reagent according to the manufacturer’s instructions. cDNA synthesis from total RNA (1 μg) was produced by reverse transcription (RT) using the superscript II kit according to the manufacturer’s protocol (Invitrogen). *Semi quantitative reverse transcription polymerase chain reaction (RT-PCR)* was performed first in pig fibroblasts and endothelial cells of mammary artery cDNAs as template to qualify the primers. The PCR reactions were performed using Taq-polymerase manufacturing protocol (Promega). The PCR program used to amplify all the tested genes consisted in a denaturing cycle of 2 min at 94°C, 35 cycles of PCR (94°C for 30s, variable annealing temperature for 30s and extensions of 72°C for 30s), and a final step at 72°C for 10 min followed by cooling at 4°C. PCR products were subjected to electrophoresis using a 2% agarose gel, stained with GelRed™ (Biotium) and illuminated with UV light.

Primer sequences and expected product lengths are shown in Supplemental Table 1.

1. ***pASC doubling time (DT)***

pASCs were plated onto 35-mm culture plates at 5x10^4^ cells/well. The cells were collected daily and fixed in formaldehyde 11.1% for eight days. The cells received a code and were counted blindly. The logarithm number (log) of cells in each of the days was calculated and plotted in an exponential graph. The linear regression was performed.

1. ***Senescence***

pASCs were fixed for 5 min in fixation solution (2% formaldehyde and 0.2% glutaraldehyde) and then incubated in freshly prepared SA-β-Gal staining solution (1 mg/mL X-Gal, 5 mM potassium ferricyanide, 5 mM potassium ferrocyanide, 150 mM NaCl, 2 mM MgCl_2_, 40 mM citric acid/phosphate buffer at pH 6.0) for 24 h at 37°C. Eight random fields were digitally photographed in a phase-contrast microscope (100X magnification). The percentage of positive blue-dyed cells was counted by blinded observer to the passages.

1. ***Karyotype***

To perform the karyotype of pASCs 1×10^4^cells/cm² were plated in 60 mm culture dishes. After 40 hours the cells were incubated with 10 μg/ml colcemid (Gibco) for 90 minutes in a humidified incubator (5% CO2, 37°C). After this the culture dishes were washed twice with PBS 1X and trypsinized. The cells were collected in 50 ml tubes and pelleted (centrifuged 1500RPM, 5 minutes, 4°C) and then incubated in 5 ml hypotonic buffer (0.1 M KCl) for 18 min at 37°C followed by fixation with methanol/glacial acetic acid (3:1) solution. Fixed cells were dropped on wet slides and air-dried per 3 days to obtain standard G-banding chromosome pattern. *Giemsa* staining was carried out as indicated by manufacturers (KaryoMAX® Giemsa, Gibco). Metaphases were fully karyotyped under a Leica HC microscope. Images were then captured with digital camera Leica DC250 and using Leica CW4000 Karyo software.

1. ***pASC plasticity - cell differentiation medium***

Adipogenic differentiation was induced by culturing pASC cells for 3 weeks in adipogenic medium with exchange every three days. (DMEM, 10% FBS, 0.5 mM isobutyl-methylxanthine (IBMX), 1 mM dexamethasone, 10 mM insulin, 200 uM indomethacin, 1% antibiotic/antimycotic).

Osteogenic differentiation was induced by culturing pASCs for 3 weeks in osteogenic medium with exchange every three days. (DMEM, 10% FBS 0.1 mM dexamethasone, 50 mM ascorbate-2-phosphate, 10 mM β-glycerophosphate, 1% antibiotic/ antimycotic). The cells were fixed for 60 min at room temperature in 70% ethanol and washed twice with distilled water. Then cells were incubated in 2% (wt/vol) Alizarin Red S reagent for 30-45 minutes at room temperature. Excess stain was removed by washing with tap water. Adipogenic and osteogenic induced cells were photographed after staining.
